# Cellular and molecular mechanisms that mediate basal and tumour necrosis factor-α-induced regulation of myosin light chain kinase gene activity

**DOI:** 10.1111/j.1582-4934.2008.00302.x

**Published:** 2008-03-17

**Authors:** Dongmei Ye, Thomas Y Ma

**Affiliations:** Department of Internal Medicine, University of New Mexico School of Medicine and Albuquerque Veterans Affairs Medical CenterAlbuquerque, NM, USA

**Keywords:** tumour necrosis factor-α, NF-κB subunit, intestinal permeability, tight junction, myosin light chain kinase

## Abstract

The patients with Crohn's disease (CD) have a ‘leaky gut’ manifested by an increase in intestinal epithelial tight junction (TJ) permeability. Tumour necrosis factor-α (TNF-α) is a proto-typical pro-inflammatory cytokine that plays a central role in intestinal inflammation of CD. An important pro-inflammatory action of TNF-α is to cause a functional opening of intestinal TJ barrier. Previous studies have shown that TNF-α increase in TJ permeability was regulated by an increase in myosin light chain kinase (MLCK) gene activity and protein expression. The major aim of this study was to elucidate the cellular and molecular mechanisms that mediate basal and TNF-α-induced increase in MLCK gene activity. By progressive 5′ deletion, minimal MLCK promoter was localized between −313 to +118 on MLCK promoter. A p53 binding site located within minimal promoter region was identified as an essential determinant for basal promoter activity. A 4 bp start site and a 5 bp downstream promoter element were required for MLCK gene activity. TNF-α-induced increase in MLCK promoter activity was mediated by NF-κB activation. There were eight κB binding sites on MLCK promoter. The NF-κB1 site at +48 to +57 mediated TNF-α-induced increase in MLCK promoter activity. The NF-κB2 site at −325 to −316 had a repressive role on promoter activity. The opposite effects on promoter activity were due to differences in the NF-κB dimer type binding to the κB sites. p50/p65 dimer preferentially binds to the NF-κB1 site and up-regulates promoter activity; while p50/p50 dimer preferentially binds to the NF-κB2 site and down-regulates promoter activity. In conclusion, we have identified the minimal MLCK promoter region, essential molecular determinants and molecular mechanisms that mediate basal and TNF-α-induced modulation of MLCK promoter activity in Caco-2 intestinal epithelial cells. These studies provide novel insight into the cellular and molecular mechanisms that regulate basal and TNF-α-induced modulation of MLCK gene activity.

## Introduction

The patients with Crohn's disease (CD) have a ‘leaky gut’ characterized by an increase in intestinal epithelial tight junction (TJ) permeability [1–9]. The defective intestinal TJ barrier has been postulated to be an important pathogenic factor contributing to intestinal inflammation of CD [1–5, 10–20]. The defect in intestin al TJ barrier allows increased paracellular permeation of luminal antigens leading to an inflammatory response [1–3, 10–12, 19]. Tumour necrosis factor-α (TNF-α) is a proto-typical pro-inflammatory cytokine that plays a central role in intestinal inflammation of CD and other inflammatory disorders [20–23]. The importance of TNF-α in intestinal inflammation of CD has been well validated by clinical and animal studies demonstrating the efficacy of anti-TNF-α antibody therapy in the treatment of moderate-to-severe active CD [23–26] and in animal models of intestinal inflammation [27–30].

A number of studies have shown that an important pro-inflammatory action of TNF-α is to cause a functional opening of intestinal TJ barrier; allowing increased paracellular permeation of noxious luminal antigens that induce inflammatory response [31–36]. TNF-α, at physiologically relevant concentrations (1–10 ng/ml), causes an increase in intestinal TJ permeability, manifested by an increase in epithelial permeability to paracellular markers and a drop in transepithelial resistance [32, 34, 37–40]. Previous studies have shown that the TNF-α-induced increase in Caco-2 intestinal epithelial TJ permeability was regulated by an increase in MLCK gene and protein expression [32, 41]; and inhibition of TNF-α-induced increase in MLCK transcription or protein expression prevented the TNF-α increase in intestinal TJ permeability.

Recent animal studies have shown that MLCK plays a central role in immune, stress or bacterial endotoxin-mediated increase in intestinal permeability and subsequent inflammatory response [28, 29, 42–44]. In these studies, the increase in intestinal permeability in mice was associated with an increase in intestinal MLCK gene and protein expression [28, 29, 42–44]; and the inhibition of MLCK activity with known pharmacologic inhibitors including ML-7 or PIK prevented the increase in intestinal TJ permeability. Moreover, the pharmacologic inhibition of MLCK activity and increase in intestinal TJ permeability prevented the subsequent development of intestinal inflammation in mice [29, 42, 44]. Human studies have also shown that patients with CD have an increase in MLCK protein expression. The increase in MLCK protein expression in CD intestinal tissue directly correlated with the level of intestinal inflammation [45]. These studies suggested that the increase in MLCK protein expression and activity contributes to the intestinal permeability increase in animals and humans; and MLCK has been identified as a potential therapeutic target to induce re-tightening of intestinal TJ barrier in inflammatory conditions [28, 29, 42–45].

In previous studies, we have identified and cloned a functionally active MLCK promoter region [46]. These studies have shown that TNF-α-induced increase in Caco-2 TJ permeability was mediated by an increase in MLCK gene activity and gene transcription, MLCK protein expression and activity [46]. However, the intracellular and molecular mechanisms that mediate the basal and TNF-α-induced increase in MLCK gene activity and subsequent increase in MLCK protein expression remain unclear. The major aim of this study was to elucidate the cellular and molecular mechanisms that regulate the basal and TNF-α-induced increase in MLCK gene activity and the subsequent increase in MLCK protein expression, using filter-grown Caco-2 intestinal epithelial cells. In the first part of this study, we identified the minimal MLCK promoter region and the essential molecular determinants that regulate the basal MLCK promoter activity in Caco-2 monolayers. In the second part, we identified the molecular determinants and the molecular processes that mediate the TNF-α-induced increase in MLCK promoter activity and protein expression. These studies provide novel insight into cellular and molecular mechanisms that mediate the basal and TNF-α-induced increase in MLCK gene activity and protein expression.

## Materials and methods

### Materials

Dulbecco's modified eagle medium (DMEM), trypsin and foetal bovine serum (FBS) were purchased from Life Technologies (Gaithersburg MD, USA). Glutamine, penicillin, streptomycin and phosphate-buffered saline solution (PBS) were purchased from Invitrogen Corporation (Carlsbad, CA, USA). TNF-α, NF-κB antibody and MLCK antibody were purchased from Sigma (St. Louis, MO, USA). [γ-^32^P]adenosine 5′-triphosphate (ATP) was from Amersham Biosciences (Piscataway, NJ, USA). ELISA reagents were from Active Motif (Carlsbad, CA, USA). Transwell permeable filters were purchased from Corning (Corning, NY, USA). Primers were from Integrated DNA Technologies (Coralville, IA, USA). NF-κB p65 siRNA was from Dharmacon, Inc. (Chicago, IL, USA). Luciferase assay reagents were from Promega (Madison, WI, USA). Transfection and cloning reagents were from Invitrogen (Carlsbad, CA, USA).

### Cell cultures

Caco-2 cells were purchased from the American Type Culture Collection at passages 18 to 20 (Rockville, MD, USA). The cells were maintained in a culture medium composed of DMEM with 4.5 mg/ml glucose, 50 U/ml penicillin, 50 U/ml streptomysin, 4 mmol/l glutamine and 10% FBS [32]. The cells were kept at 37°C in a 5% CO_2_ environment. Culture medium was changed every 2 days. The cells were subcultured by partial digestion with 0.25% trypsin and 0.9 mmol/l ethylenediaminetetraacetic acid (EDTA) in Ca^2+^-free and Mg^2+^-free PBS.

### MLCK promoter reporter constructs

Construction of MLCK promoter reporter plasmids was carried out using the pGL-3 basic luciferase reporter vector. Deletions of MLCK promoter were done by the PCR method. The primers used for cloning various MLCK promoter deletion constructs are listed in Table 1. The PCR conditions were 1 cycle at 94°C for 2 min., followed by 43 cycles at 94°C for 1 min., 50°C for 1 min. and 72°C for 2 min., 1 cycle at 72°C for 5 min. The resultant PCR products were cloned into pGL-3 basic luciferase reporter vector and the sequences were verified by DNA services at University of New Mexico.

**Table 1:** Sequences of cloning primers

Primer name	Sequence (5′∼ 3′)
FL-MLCK(+)	GCCGGTACCGAGAAGCAGGAGAGTATTAAATG
FL-MLCK(−)	CCAAGCTTATGTTTGTTGTGGCAACTGGGC
MLCK-1628(+)	CGGGGTACCTCTTCAAGGTCAAGAGATGTC
MLCK-929(+)	CGGGGTACCCTCTGCCCTCTTGACTTAATC
MLCK-509(+)	GATGGTACCTGAAGGTAGGAGAGACACTC
MLCK-313(+)	GCCGGTACCATGGCCTTCCTCCCTCACCCCT
MLCK-245(+)	CGGGGTACCTGAGTACCACCGTCATGGTTC
MLCK-76(+)	CGGGGTACCTGTCCCAGAGTGATGTACTCC
MLCK+46(+)	GCCGGTACCAGTTCAGAGCAACTTCAGGAG
MLCK+66(+)	GCCGGTACCAGAGCTTCAGGACGCCTTTC

### Site-directed mutagenesis

Mutagenesis of MLCK promoter were performed by using the GeneTailor Site-Directed Mutagenesis System (Invitrogen). Briefly, primers (sequences in Table 2) were generated that included the mutation site flanked by a wild-type sequence on either side. A PCR reaction produced a new complete copy of the plasmid containing the mutation coded for by the primers. The linear PCR product was subsequently transformed into DH5-T1 *Escherichia coli*, which circularized the PCR product and digested any remaining parent plasmid. DNA sequence was verified by DNA services at University of New Mexico.

**Table 2:** Sequences of mutagenesis primers

Primer name	Sequence (5′∼ 3′)
P53(+)	CCCATGGCCTTCCTCCCTCATTTTCATTGAAATTCTCTTTTAGACCCTGC
P53(−)	TGAGGGAGGAAGGCCATGGGTACCTATCGA
TSS-8bp(+)	GGCTTTCCTTTCCTCCTTCCCAGGTGGCTC
TSS-8bp(−)	GAAGGAGGAAAGGAAAGCCAAATCAGAGA
TSS-4bp(+)	CTTTCCTTTCCTCCTTCCCACTTGTCCAGGTGGCTC
TSS-4bp(−)	GTGGGAAGGAGGAAAGGAAAGCCAAATCAGA
DPE(+)	GGCTGCCTCTGCTGCAGTTCGAGATAACTTCAGGA
DPE(−)	GAACTGCAGCAGAGGCAGCCGGGAGCCACC
NF- B1-Mu(+)	TGCTGCAGTTCAGAGCAACTCTGAAGATCCTTTGACCGAGAGCTTC
NF- B1-Mu(−)	AGTTGCTCTGAACTGCAGCAGAGGCAGCCG

### Nuclear extracts, electrophoretic mobility shift assay (EMSA) and ELISA

Filter grown Caco-2 cells were treated with 10 ng/ml of TNF-afor 30 min. Cells were washed with 1 ml of ice-cold PBS, scraped and centrifuged at 14,000 RPM for 30 sec. The cell pellets were re-suspended in 200 μl of buffer A (10 mM Hepes-KOH, 1.5 mM MgCl_2_, 10 mM KCl, 0.5 mM dithiothreitol (DTT) and 0.2 mM phenylmethanesulfonyl fluoride (PMSF) pH = 7.9), and incubated on ice for 15 min. After centrifugation at 14,000 RPM for 30 sec, pelleted nuclei were re-suspended in 30 μl of buffer C (20 mM Hepes-KOH, 25% glycerol, 420 mM NaCl, 1.5 mM MgCl_2_, 0.2 mM EDTA, 0.5 mM DTT and 0.2 mM PMSF, pH = 7.9). Following incubation on ice for 20 min., the lysates were centrifuged at 14,000 RPM for 20 min. The supernatants were stored at −70°C. Protein concentrations were determined using the Bradford method. Two double-stranded oligonucleotides (Integrated DNA Technologies) were designed to encode the NF-κB1 and 2 binding sites. The oligonucleotides were end-labelled with [γ-^32^P] ATP using T4 polynucleotide kinase (Promega). The binding reactions contained 10 μg of proteins, 1 ng of labelled probe and 4 μl of gel shift binding buffer with a total volume of 20 μl. For competition experiment, unlabelled probe was added to the reaction mixture in 100-fold excess prior to adding the labelled probe. After incubation at room temperature for 30 min., the reaction mixtures were elec-trophoresed on a 4% polyacrylamide gel in 0.5× tris-base EDTA (TBE) buffer. Gels were subsequently dried and developed using a phosphorimager (Storm Scanner, Amersham Biosciences, Piscataway, NJ, USA). To determine which NF-κB subunit binds to NF-κB1 or 2 binding site, ELISA studies were performed using TransAM NF-κB family Flexi kit (Active Motif). In brief, the binding reactions contained 1 pmol of biotinylated probe (Integrated DNA Technologies) and 5 μg of nuclear extract in complete binding buffer with a total volume of 50 μl. After 30 min. of incubation, the solution was transferred to an individual well on the plate, and incubated for 1 hr. 100 μl rabbit NF-κB p65 or p50 antibody was added to the well to bind NF-κB subunits from the nuclear extract. After incubation for 1 hr, antibodies were removed and 100 μl anti-rabbit HRP-con-jugated IgG was added to the well and incubated for 1 hr. Subsequently 100 μl developing solution was added for 2–10 min., and 100 μl stop solution added. The absorbance at 450 nm was determined using the SpectrraMax 190 (Molecular Devices, Sunnyvale, CA, USA).

### Transfection of DNA constructs

DNA constructs of MLCK promoters were transiently transfected into Caco-2 cells using transfection reagent lipofectamine 2000 (Life Technologies, Inc.). Renilla luciferase vector (pRL-TK, Promega) was co-transfected with each plasmid construct as an internal control. 5 **×** 10^5^ cells/filter were seeded into a 6-well transwell plate and grown to confluency. Caco-2 monolayers were then washed with PBS twice and 1.0 ml of Opti-MEM medium was added to the apical compartment of each filter and 1.5 ml was added to the basolateral compartment of each filter. 1 μg of each plasmid construct and 0.25 μg of pRL-TK or 2 μl of lipofectamine 2000 were pre-incubated in 250 μl of Opti-MEM, respectively. After 5 min. of incubation, two solutions were mixed and incubated for another 20 min., and the mixture was added to the apical compartment of each filter. After incubation for 3 hrs at 37°C, 500 μl of DMEM containing 10% FBS and no antibiotics was added to both sides of the filter to reach a 2.5% final concentration of FBS. Subsequently, media were replaced with normal Caco-2 growth media 16 hrs after transfection. The experiments were carried out 48 hrs after transfection.

### Luciferase assay

After the TNF-α treatment (7 hrs) [47], Caco-2 cells were washed twice with 1 ml of ice-cold PBS, followed by the addition of 400 μl of 1× passive lysis buffer, incubated at room temperature for 15 min., scraped and transferred into an eppendorf tube and centrifuged for 15 sec. at 13,000 RPM in a micorcentrifuge. Luciferase activity was determined using the dual luciferase assay kit (Promega). 20 μl of the supernatant was used for each assay. Luciferase values were determined by Lumat LB 9507 (EG&G Berthold, Oak Ridge, TN, USA). The value of reporter luciferase activities were then divided by that of renilla luciferase activities to normalize for differences in transfection efficiencies. The average activity value of the control samples was set to 1.0. The luciferase activity of MLCK promoter in treated samples was determined relatively to the control samples.

### siRNA of p53 and NF-kB p65

Smart pool of p53 and NF-κB siRNA was purchased from Dharmacon, Inc. (Chicago, IL, USA). Caco-2 monolayers were transiently transfected using DharmaFect transfection reagent (Lafayette, CO). Caco-2 cells were seeded into a 6-well plate and grown to confluency. Caco-2 monolayers were then washed with PBS and Opti-MEM medium was added to the well. P53 or NF-κB siRNA and DharmaFect reagent were pre-incubated in Opti-MEM. After 5 min. of incubation, two solutions were mixed and incubated for another 20 min., and the mixture was added to each well. After incubation for 3 hrs at 37°C, 500 μl DMEM containing 10% FBS and no antibiotics were added to cell culture media to reach a 2.5% final concentration of FBS. TNF-α treatment was carried out 96 h post transfection. The siRNA-induced silencing of NF-κB p65 was confirmed by immunoblot of NF-κB p65.

### Western blot

MLCK and NF-κB p65 protein expressions were assessed by western blot as previously described [32]. Filter-grown Caco-2 monolayers were serum deprived overnight. Caco-2 monolayers were then treated with 10 ng/ml TNF-α for desired time periods. Cells were washed twice with cold PBS, and lysed with 500 μl lysis buffer (Lysis Buffer: 50 mM Tris-HCl pH 7.5, 100 mM NaCl, 50 mM NaF, 5 mM EDTA, 40 mM β-glycerophosphate, 200 μm vanadate, 100 μm PMSF, 1 μg/ml leupeptin, 1 μg/ml pepsattin-A, 40 mM p-nitrophenyl phosphate (PNPP), 1 μg/ml aprotinin and 0.5% NP-40), and scraped, then centrifuged for 5 min. at 7000 ×*g* to yield a clear lysate. Protein concentration was determined by Lowry method. 10 μg of protein from each sample was loaded into a SDS-PAGE gel. The gel was transblotted against anti-MLCK or anti- NF-κB p65 antibody.

### Determination of Caco-2 epithelial monolayer resistance

The effect of TNF-α on Caco-2 monolayer epithelial electrical resistance was measured using epithelial volt-ohmmeter (World Precision Instruments, Sarasota, FL, USA) as previously described [48, 49]. For resistance measurements, both the apical and basolateral sides of the epithelia were bathed in regular growth media. Electrical resistance was measured until similar values were recorded on three consecutive measurements. Each experiment was repeated at least three times in quadruplicate to ensure reproducibility.

### Statistical analysis

The values of experimental data were expressed as the mean ± S.E., and analysed using two-tailed unpaired t-test (Graph Pad Prizm 4.00 for Windows, GraphPad Software, San Diego, CA, USA). *P*-values of <0.05 were considered significant. All experiments were repeated at least three times to ensure reproducibility.

## Results

### Genomic structure of MLCK

**Fig. 1:**
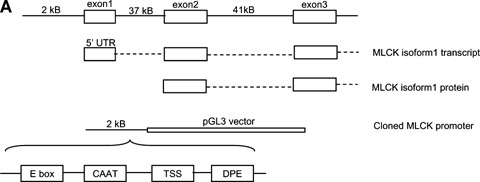

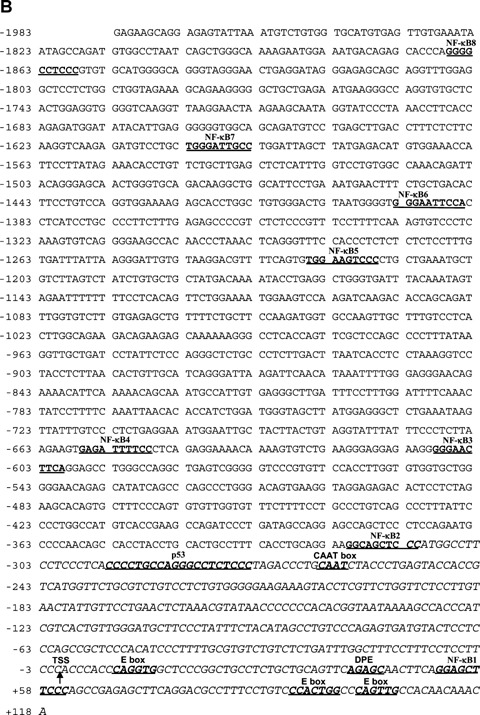
Genomic structure of MLCK isoform 1 and DNA sequence of cloned MLCK promoter region. (**A**) Schematic diagram of MLCK genomic structure. MLCK translation start site is located in exon 2. Transcription start site and 5′ UTR are located in exon 1. Approximately 2 kB promoter region upstream of exon 1 was cloned into pGL3 basic vector. (**B**) DNA Sequence of the cloned 2091 bp MLCK promoter region. Minimal promoter region are in italic. Transcription factor binding sites are in bold, underlined and labelled. Transcription start site is labelled with an arrow.

MLCK is expressed as eight isoforms, including non-muscle and smooth muscle isoforms and a kinase-related protein telokin, which utilize different transcription start sites within a single gene. MLCK isoforms 1 and 2 are expressed in human intestinal epithelial cells [50]. MLCK isoform 1 localizes to the intestinal epithelial TJs and is involved in the regulation of TJ barrier function. MLCK isoform 1 is a full-length transcript and encodes the full-length non-muscle isoform. To determine the genomic structure of MLCK isoform 1, the human genome database was searched with MLCK isoform 1 cDNA (Genbank accession No NM 053025). BLAST analysis showed that MLCK isoform 1 is mapped to chromosome 3q21 region and organized into 32 exons. The translation start site is located in exon 2. The exon 1 and exon 2 are separated by a 37.7 kb intron. The transcription start site and 5′ untranslated region are located in exon 1 (Fig. 1A).

The sequence of MLCK promoter has no TATA box near the transcriptional initiation site. However, a CAAT box and several E boxes are located within the minimal promoter region. Also, a downstream promoter element (DPE) AGAGC which normally resides in TATA less promoter is located at +36 to +40 [51]. These findings suggested that the regulatory elements required for the gene transcription were present in this region (Fig. 1A).

### Identification of MLCK minimal promoter region

**Fig. 2:**
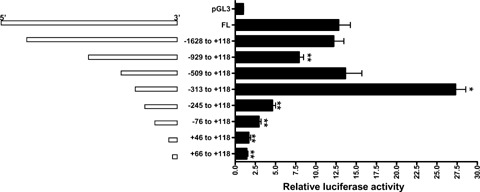
Identification of MLCK minimal promoter region. Progressive increasing 5′ deletion constructs of full-length (FL) MLCK promoter were generated. Filter-grown Caco-2 cells were transfected with these constructs and promoter activities were determined by luciferase assay as described in the Materials and methods section. Data are represented as mean of four replicates ± S.E. *P**<** 0.05 compared to control. **P < 0.05 compared to MLCK −313 to +118.

Previously, we cloned a 2091 bp MLCK promoter region (Genbank accession number DQ 090939) into a pGL3 basic vector [46]. The sequence of the full-length (FL) 2091 bp MLCK promoter region is shown in Figure 1B. In the following studies, we determined the minimal promoter region. For this purpose, a progressively increasing 5′ deletion constructs were generated (total of eight) and sub-cloned into the pGL3 basic vector (Fig. 2). Deletion constructs were then transfected into confluent Caco-2 cells and the promoter activity determined by luciferase assay (Fig. 2). As shown in Fig. 2, progressive deletion of 5′ end extending to −314 did not result in a significant increase in promoter activity compared to the FL promoter (2091 bp). The deletion construct MLCK-313 exhibited a 2-fold increase in promoter activity compared to the FL promoter (Fig. 2). The deletion of 5′ end extending beyond −246 resulted in a marked decrease in promoter activity. Based on these results, we identified −313 to +118 as a minimal MLCK promoter region.

### Regulation of basal MLCK promoter activity

**Fig. 3:**
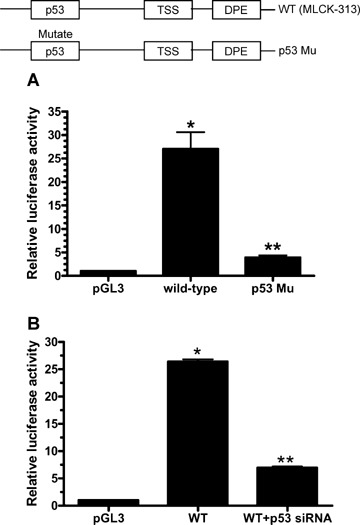
p53 binding site is essential for basal MLCK expression. (**A**) P53 binding site located within minimal promoter region (−294 to −275) was mutated and promoter activity determined by luciferase assay as described in the Materials and methods section. (**B**) Effect of p53 siRNA transfection on basal MLCK promoter activity. Transfection of Caco-2 cells with p53 siRNA resulted in a significant decrease in basal MLCK −313 (WT) promoter activity. Data are represented as mean of four replicates ± S.E. *P < 0.05 compared to pGL3. **P < 0.05 compared to wild-type.

As shown in Figure 2, extending the deletion to include 68 bp MLCK promoter region between −313 to −245 resulted in a marked decrease in promoter activity. These findings suggested that a regulatory site within this 68 bp region could have a critical role in the maintenance of basal MLCK promoter activity. Using the Genomatix/Promoter Inspector software, a p53 transcription factor binding motif (−294 to −275) was identified within this 68 bp region (Fig. 1B). To determine the possible regulatory function of this p53 binding motif (CCCCTGCCAGGGCCTCTCCC) on basal promoter activity, the p53 binding site was mutated via site-directed mutagenesis in the construct MLCK-313 (which encodes the minimal promoter region). The mutation of p53 site (−294 to −275) resulted in a near complete inhibition of promoter activity (Fig. 3). It should be noted that the basal promoter activity of the p53 mutant MLCK promoter was similar to deletion construct MLCK −245 (which lacks the p53 binding region) (Fig. 2), indicating that p53 binding region has a critical role in the regulation of basal MLCK promoter activity. Thus, the sharp drop in promoter activity between MLCK −313 and MLCK −245 observed in Fig. 2 could be explained by the absence of p53 site. To further substantiate the role of p53 in basal promoter activity, p53 expression was knockdown by p53 siRNA transfection of Caco-2 monolayers. The p53 siRNA transfection also resulted in a marked decrease in basal MLCK promoter activity (Fig. 3B), confirming that p53 plays an important role in the regulation of basal MLCK promoter activity.

**Fig. 4:**
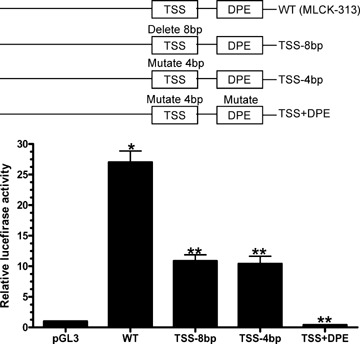
Effect of transcription start site (TSS) or downstream promoter element (DPE) site mutation on minimal MLCK promoter activity. Constructs containing either 8 bp deletion or 4 bp mutation of TSS and double mutation of TSS and DPE sites were generated as described in the Materials and methods section. Promoter activities were assessed by luciferase assay. Data are represented as mean of four replicates ± S.E. *P < 0.05 compared to pGL3. **P < 0.05 compared to wild-type.

Next, the functional activity of the transcription start region was experimentally validated by selective deletion and site-directed mutagenesis. Using MLCK −313 (which encompasses the minimal promoter region) as the ‘wild-type’ template, a deletion construct lacking the eight base pair −6 to +2 (CCACCCAC) start region was generated (Fig. 4). The deletion of 8 bp start sequence resulted in a significant decrease in luciferase activity (Fig. 4). To further narrow the start site, a four base pair mutant which replaces CCAC (−2 to +2) with TTGT was generated. The 4 bp mutation also resulted in a similar decrease in luciferase activity as the 8 bp deletion construct (Fig. 4), suggesting that transcription start site (TSS) was located within this four base pair sequence. To determine the role of DPE in basal gene transcription process, a double mutation of the TSS and DPE was generated. The double mutation resulted in almost complete absence of the promoter activity (Fig. 4), confirming that these two sites were essential for the initiation of MLCK gene transcription.

### NF-κB regulation of MLCK promoter activity

**Fig. 5:**
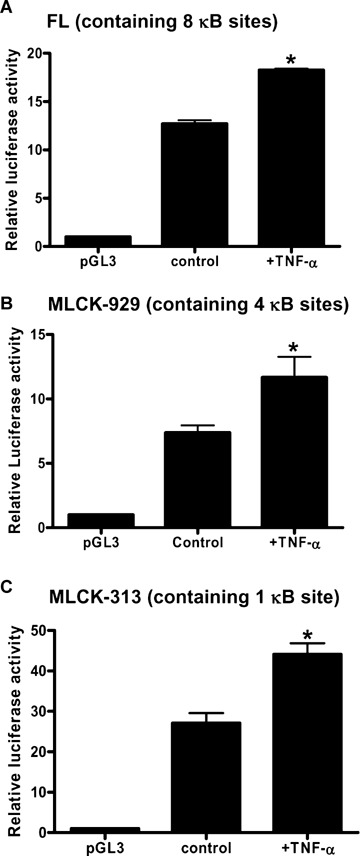
TNF-α (10 ng/ml) effect on MLCK promoter activity in deletion constructs containing decreasing numbers of NF-κB sites. (**A**) FL MLCK promoter (containing 8 κB sites); (**B**) MLCK -929 (containing 4 κB sites); (**C**) MLCK -313 (containing only the NF-κB1 site). Data are represented as mean of four replicates ± S.E. *P < 0.05 compared to control.

**Fig. 6:**
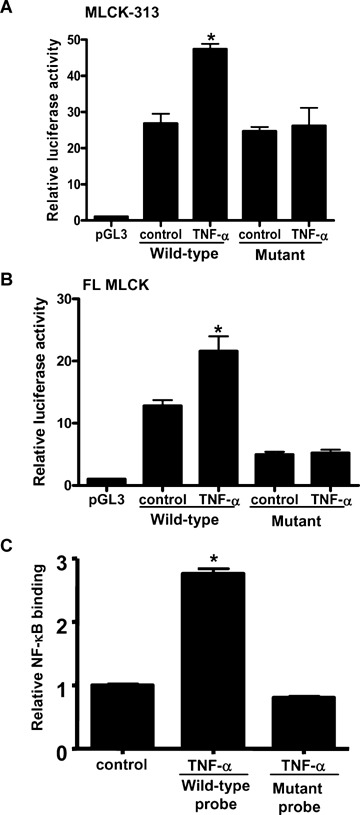
NF-κB1 binding site on MLCK promoter is required for the TNF-α-induced increase in MLCK promoter activity. (**A**) TNF-α effect on MLCK promoter activity in promoter construct MLCK −313 having either intact or mutated NF-κB1 binding site. (**B**) TNF-α effect on FL MLCK promoter activity in promoter construct having either intact or mutated NF-κB1 binding site. (**C**) TNF-α effect on NF-κB binding to DNA probe having either wild-type or NF-κB1 mutated binding site. 50 bp probes containing the sequence of wild-type or mutated NF-κB1 binding site on MLCK promoter were synthesized as described in the Materials and methods section. ELISA was used to determine the binding. Data are represented as mean of three replicates ± S.E. *P**<** 0.05 compared to control.

Previously, we reported that TNF-α-induced increase in MLCK gene transcription was mediated by nuclear transcription factor NF-κB [32]. In the following studies, we expanded on our previous findings to delineate the specific molecular determinants on MLCK promoter that mediated the NF-κB action. Eight putative NF-κB binding sites were identified on MLCK promoter using the Genomatix/Promoter Inspector software. The location of each of the eight NF-κB binding sites is shown in Figure 1B. Note that seven κB sites (NF-κB2 to 8 sites) were located outside the minimal promoter region and one κB site (NF-κB1 site) was located within the minimal region. To delineate the specific κB sites that mediated the TNF-α-induced up-regulation of MLCK promoter activity, the TNF-α effect on promoter activity of deletion constructs having progressively larger 5′ end deletion was determined. As shown in Fig. 5, TNF-α caused a significant increase in MLCK promoter activity in the FL MLCK promoter construct compared to the control (*P* < 0.05). TNF-α also caused a similar proportional increase in MLCK promoter activity in the deletion constructs lacking the NF-κB2 to 8 sites (Fig. 5B and C), indicating that NF-κB2 to 8 sites were not necessary for the TNF-α-induced up-regulation of MLCK promoter activity. These findings suggested that the NF-κB1 site (located within the minimal region) may be the regulatory site that mediated the TNF-α-induced up-regulation of MLCK promoter activity. To test this possibility, NF-κB1 motif was mutated in MLCK-313. In the wild-type MLCK-313, TNF-α caused an increase in MLCK promoter activity (Fig. 6A). The mutation of the NF-κB1 site completely prevented the TNF-α-induced increase in promoter activity (Fig. 6A). In separate studies, the effect of NF-κB1 mutation on FL MLCK promoter activity was also determined. The mutation of the NF-κB1 site in the FL MLCK also prevented the TNF-α-induced increase in promoter activity (Fig. 6B). These results indicated that the NF-κB1 site was required for the TNF-α-induced increase in promoter activity.

Next, the binding of TNF-α activated NF-κB to the NF-κB1 binding site on MLCK promoter region was determined using an ELISA-based DNA-binding assay. In these studies, 50 bp double-stranded DNA probe encoding the NF-κB1 site (30 bp up- and 10 bp downstream of NF-κB1 site) on MLCK promoter region was synthesized and used as a probe to assess the binding of NF-κB to the NF-κB1 motif. The nuclear extracts from control and TNF-α treated Caco-2 monolayers were used to assess the binding of NF-κB to the DNA probe. TNF-α treatment resulted in a significant increase in NF-κB binding to the NF-κB1 binding site on the DNA probe (Fig. 6C). The mutation of NF-κB1 binding site on DNA probe prevented the TNF-α increase in NF-κB binding to the DNA probe (Fig. 6C). These findings confirmed that TNF-α causes an increase in NF-κB binding to the NF-κB1 binding motif, which presumably leads to the activation of MLCK promoter.

### p53 binding site does not play a role in TNF-α-induced increase in MLCK promoter activity

**Fig. 7:**
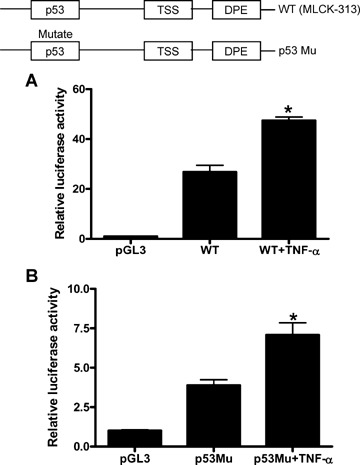
Effect of p53 mutation on TNF-α-induced increase in MLCK promoter activity. (**A**) TNF-α effect on MLCK -313 promoter activity. Caco-2 cells were transfected with MLCK −313, promoter activity was determined by the luciferase assay and expressed as relative luciferase activity as described in the Materials and methods section. Data are represented as mean of three replicates ± S.E. *P**<** 0.05 compared to WT. (**B**) TNF-α effect on promoter activity of MLCK −313 with a mutated p53 binding site. Data are represented as mean of four replicates ± S.E. *P < 0.05 compared to p53 Mu.

Above studies indicated that the p53 site (−294 to −275) plays a crucial role in basal MLCK promoter activity. The possible involvement of this site in mediating the TNF-α modulation of MLCK promoter activity was also examined by site-directed mutagenesis of p53 binding motif. The mutation of p53 binding site did not inhibit the TNF-α-induced increase in MLCK promoter activity (Fig. 7). Although the basal promoter activity in the p53 mutant promoter was lower, there was a similar proportional increase in MLCK promoter activity in response to TNF-α treatment in both the WT MLCK −313 and p53 mutant constructs. These findings suggested that although p53 site plays a crucial role in the regulation of basal promoter activity, this site was not involved in mediating the TNF-α regulation of MLCK promoter activity.

### Molecular mechanisms of TNF-α regulation of MLCK promoter activity

**Fig. 8:**
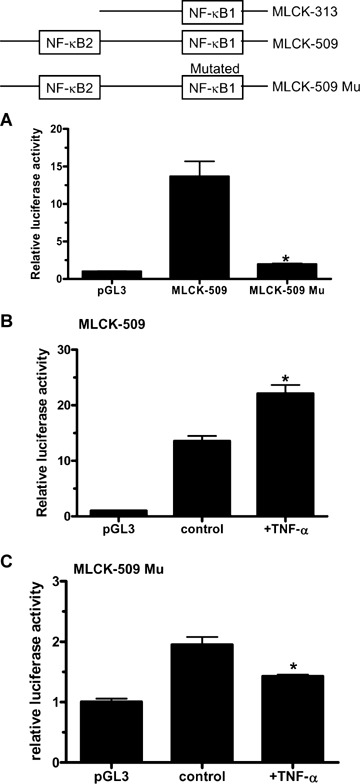
Effect of NF-κB1 site mutation on basal and TNF-α-induced increase in promoter activity. (**A**) Comparison of basal promoter activity of MLCK −509 and MLCK −509 having mutant NF-κB1 site (MLCK −509 Mu). (**B**) TNF-α effect on MLCK −509 construct. (**C**) TNF-α effect on MLCK −509 construct containing the NF-κB1 site mutation (MLCK −509 Mu). Data are represented as mean of four replicates ± S.E. *P < 0.05 compared to control.

To delineate the molecular mechanisms involved in NF-κB regulation of MLCK promoter activity, the role of NF-κB1 and 2 sites in the regulation of MLCK promoter activity was examined. As shown in Fig. 2, deletion of six upstream NF-κB sites (NF-κB3 to 8) between −1973 and −509 did not affect the basal MLCK promoter activity. However, extending the deletion to include NF-κB2 site between −325 and −316 resulted in a 2-fold increase in basal promoter activity (Fig. 2), suggesting that the NF-κB2 site may have a repressive effect on promoter activity. To investigate the possible interactive relationship between NF-κB1 and 2 site, the effect of NF-κB1 site mutation in the presence of NF-κB2 site was determined using MLCK −509 (which contains the NF-κB1 and 2 sites) (Fig. 8). The mutation of NF-κB1 site resulted in a marked decrease in MLCK −509 promoter activity (Fig. 8A). These results suggested that NF-κB1 and 2 sites may have opposite regulatory actions on MLCK promoter activity; NF-κB2 site appears to suppress while NF-κB1 site appears to up-regulate the MLCK promoter activity. To further examine this issue, subsequent studies, the regulatory response of NF-κB1 and 2 sites to TNF-α stimulation was examined. TNF-α treatment resulted in a significant increase in promoter activity of MLCK −509. In mutant MLCK −509 (with mutated NF-κB1 site), TNF-α treatment resulted in a decrease in MLCK promoter activity, suggesting that in response to TNF-α treatment NF-κB2 site down-regulates the promoter activity.

**Fig. 9:**
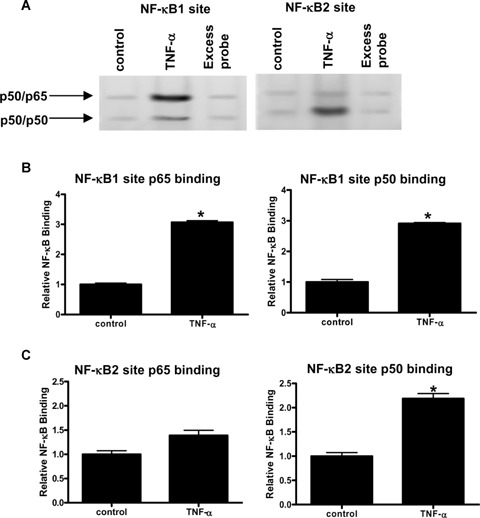
Effect of TNF-α on NF-κB dimer binding to the NF-κB1 or 2 binding site on MLCK promoter region. NF-κB dimer binding was assessed by elec-trophoretic mobility shift assay (EMSA) or ELISA-based DNA-binding assay as described in the Materials and methods section. Following TNF-α treatment for 30 min., the nuclear extracts were obtained from lysed Caco-2 monolayers and assessed for DNA binding. (**A**) Twenty-five bp oligonucleotide encoding the NF-κB1 or 2 binding site was radiolabelled and used as a DNA probe. Excess cold oligonucleotide (100-fold) was added as competitor. The representative EMSA gel showing p50/p65 and p50/p50 dimer binding was presented. (The experiments were repeated four times to ensure repro-ducibility.) (**B** and **C**) The binding of the NF-κB subunit p65 and p50 to NF-κB1 site (**B**) or NF-κB2 site (**C**) was determined by ELISA-based DNA-binding assay using p65 and p50 antibodies as described in the Materials and methods section. The binding of each NF-κB subunit to the binding sites was expressed as relative NF-κB binding activity. Data are represented as mean of three replicates ± S.E. *P < 0.05 compared to control.

Previous studies have shown that different NF-κB dimers have varying regulatory action on gene regulation. NF-κB p50/p65 dimers are known to activate gene activity while p50/p50 dimers have been shown to repress gene activity [52–55]. To investigate the molecular mechanisms responsible for the differential regulation of NF-κB1 and 2 sites, we examined the binding of specific NF-κB dimer types to the κB binding sites *via* electrophoretic mobility shift assay (EMSA) and ELISA-based antibody labelling studies. In EMSA studies, we used 25 bp oligonucleotide probes encoding either the NF-κB1 or 2 site plus 7–8 bp up- and downstream of the κB binding motifs. (The sequence of the oligonucleotide probes are: 5′-CAACTTCAGGAGCTTCCCAGCCGAG-3′ corresponding to the NF-κB1 region and 5′-TGCAGGAAG-GCAGCTCCCATGGCCT-3′ corresponding to the NF-κB2 region). As shown in Fig. 9A, TNF-α (10 ng/ml) treatment resulted in a marked increase in NF-κB p50/p65 heterodimer binding to ^32^P-labelled NF-κB1 DNA probe. There was only a small increase in p50/p50 dimer binding. In contrast, p50/p50 dimer was the predominant dimer-type binding to the NF-κB2 site in response to TNF-α treatment (Fig. 9A). These findings indicated that p50/p65 was the predominant dimer-type binding to the NF-κB1 site while p50/p50 was the predominant dimer-type binding to the NF-κB2 site following TNF-α stimulation of Caco-2 cells. The specificity of NF-κB dimer binding to the ^32^P-labelled probes was confirmed by addition of excess (100-fold higher concentration) ‘cold’ or non-radioactive labelled probe. The excess of non-radiolabelled probes inhibited the NF-κB dimer binding to the ^32^P-labelled probes (Fig. 9A). To further validate the above EMSA results, ELISA-based antibody labelling studies were also carried out. In these studies, two 50 bp DNA probes were synthesized, encoding either the NF-κB1 or 2 site plus 30 bp up- and 10 bp downstream of the κB motif. TNF-α treatment resulted in a similar proportional increase in both p65 and p50 binding to the NF-κB1 site (Fig. 9B). In contrast, TNF-α caused a significant increase in p50 binding but did not cause an increase in p65 binding to the NF-κB2 site (Fig. 9C). Together, these results indicated that in response to TNF-α treatment, p50/p65 dimer was the predominant dimer-type binding to the NF-κB1 site, while p50/p50 dimer was the major dimer-type binding to the NF-κB2 site. These results suggested that p50/p65 dimer binding to the NF-κB1 site up-regulates the promoter activity, while p50/p50 binding to the NF-κB2 site down-regulates the promoter activity [52–55].

**Fig. 10:**
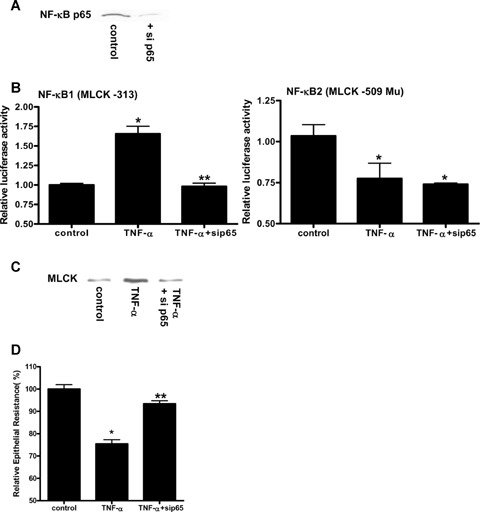
Effect of NF-κB p65 knock-down by siRNA transfection on MLCK promoter activity, protein expression and transepithelial resistance (TER). (**A**) Effect of NF-κB p65 siRNA transfection on NF-κB p65 expression in Caco-2 monolayers (96 hrs post-transfection). (**B**) Effect of siRNA knock-down of NF-κB p65 expression on TNF-α modulation of promoter activity in MLCK −313 and MLCK −509 Mu. Data are represented as mean of four replicates ± S.E. *P**<** 0.05 compared to control. **P**<** 0.05 compared to TNF-α treatment. (**C**) Effect of p65 siRNA transfection on TNF-α-induced increase in MLCK protein expression as determined by western-blot. (**D**) Effect of p65 siRNA transfection on TNF-α-induced drop in Caco-2 monolayer TER. Data are represented as mean of three replicates ± S.E. *P**<** 0.05 compared to control. **P < 0.05 compared to TNF-α.

Based on the above results, we hypothesized that the TNF-α-induced increase in MLCK promoter activity and subsequent increase in MLCK gene and protein level were regulated by NF-κB p50/p65 dimer binding to the NF-κB1 site. To validate the role of NF-κB p50/p65 dimer on MLCK promoter activation, NF-κB p65 expression was silenced in Caco-2 monolayers by NF-κB p65 siRNA transfection. As shown in Figure 10A, p65 siRNA transfec-tion resulted in a near-complete depletion of NF-κB p65 in Caco-2 monolayers. NF-κB p65 silencing resulted in a complete inhibition of TNF-α-induced increase in MLCK promoter activity in MLCK −313 (Fig. 10B), indicating that NF-κB p65 was required for the NF-κB1 site up-regulation of promoter activity. In contrast, NF-κB p65 siRNA did not affect the TNF-α-induced decrease in promoter activity in MLCK −509 Mu (which contains functionally active NF-κB2 site, but a mutated NF-κB1 site) (Fig. 10B), confirming that p50/p65 was not involved in the NF-κB2 site down-regulation of promoter activity. The NF-κB p65 silencing also inhibited the TNF-α-induced increase in MLCK protein expression, indicating that p50/p65 mediated the TNF-α increase in MLCK protein expression (Fig. 10C). Moreover, NF-κB p65 silencing also prevented the TNF-α-induced drop in Caco-2 transepithelial resistance (Fig. 10D). Together, these results indicated that NF-κB p50/p65 binding to the NF-κB1 site causes an up-regulation of MLCK gene activity, which leads to an increase in MLCK protein expression and functional opening of the Caco-2 TJ barrier.

## Discussion

Previous studies from our laboratory have shown that the TNF-α-induced increase in intestinal epithelial TJ permeability was regulated in part by NF-KB-induced increase in MLCK gene activity and MLCK protein expression [41, 46]. These studies indicated that TNF-α causes a rapid activation of NF-κB resulting in a cytoplas-mic-to-nuclear translocation of NF-κB. The activated NF-κB binds to its binding site on the MLCK promoter and causes a sequential increase in MLCK gene activity, MLCK protein expression and MLCK activity, and opening of TJ barrier [41, 46]. Although previous studies have shown that MLCK gene activation was a key intracellular process mediating the TNF-α-induced increase in MLCK protein expression/activity [46], the molecular mechanisms and the intracellular determinants that regulate MLCK gene activity remain unclear. In the present study, we extended on our previous observations and investigated the cellular and molecular mechanisms that mediate the basal and TNF-α-induced regulation of MLCK gene activity. Our studies have for the first time localized the minimal MLCK promoter region to −313 to +118 and identified p53 as a key transcription factor regulating the basal promoter activity. Our studies also delineated the TSS and the DPE that were essential for the initiation of MLCK gene transcription. Additionally, using computerized software we identified eight κB sites on the MLCK promoter region and delineated the specific κB site responsible for mediating the TNF-α-induced up-regulation of MLCK promoter activity. Using combination of molecular approaches, we were also able to provide mechanistic insight into the molecular interactions that mediate the TNF-α regulation of MLCK promoter activity.

The NF-κB family of transcription factors is known to regulate a wide range of biological activities, including inflammatory responses, cellular proliferation and apoptosis [56]. The NF-κB family consists of p50, p65, RelB, cRel and p52 subunits. These five NF-κB subunits form various combinations of dimers that mediate NF-κB dependent transcriptional regulation [57]. The p50/p65 and p50/p50 dimers are the most common dimer types in cells [58–60]. It has been shown that p50 and p65 subunits are ubiquitously expressed in various cell types [58–60]. The p50/p65 is the predominant dimer type (>90%) and p50/p50 is a minor dimer type present in cells [61–63]. The basal expression of other family members varies greatly and their presence depends on the specific cell type.

In this study, we examined the regulatory actions of two **κ**B sites; within (NF-κB1: +48 to +57) and external (NF-κB2: −325 to −316) to the minimal promoter region (Fig. 1B). Interestingly, our data indicated that the two κB sites had opposite effects on promoter activity (Fig. 8). To provide molecular explanation for the opposite effects, we examined the possibility that different NF-κB dimer types may be binding to the κB sites. The NF-κB subunit-binding studies indicated that p50/p65 preferentially binds to the NF-κB1 site, while p50/p50 preferentially binds to the NF-κB2 site (Fig. 9). Based on these findings, we hypothesized that p50/p65 binding to the NF-κB1 site induces an activation of promoter activity, while p50/p50 binding to the NF-κB2 site causes a repression of the promoter activity. In support of such possibility, previous studies have shown that p65 subunit contains a potent transacti-vation domain that leads to the activation of gene promoter region [64, 65]. In contrast, p50 lacks the transactivation domain and functions only in DNA binding [64]. The p50/p50 dimers have been shown to act as a negative regulator of NF-κB activity by competing with p50/p65 for the NF-κB response elements [65]. The p50/p50 homodimers preferentially bind to the GC rich binding sites in comparison to p50/p65 dimers [66]. Consistent with this, our data showed that p50/p50 dimers have higher affinity to the NF-κB2 site (GGCAGCTCCC) (which has higher GC content) than the p50/p65 dimers. Correspondingly, p50/p65 dimers had higher affinity to the NF-κB1 site (GGAGCTTCCC) (which has lower GC content) than the p50/p50 dimers. The mutation and siRNA knock-down studies validated the hypothesis that p50/p65 binding to the NF-κB1 site causes an increase in MLCK promoter activity, while p50/p50 binding to the NF-κB2 site causes a decrease in MLCK promoter activity.

The net result of TNF-α stimulation of Caco-2 cells is to activate MLCK promoter activity, which in turn leads to an increase in MLCK protein expression and increase in TJ permeability in Caco-2 cells (Fig. 10) [46]. Our data suggested an interesting possibility that during quiescent (unstimulated) conditions, the NF-κB2 site may have a repressive action on MLCK promoter activity (which leads to an enhancement of TJ barrier function). In con-strast, during TNF-α stimulation, activated p50/p65 dimers bind to the NF-κB1 site, causing an increase in MLCK promoter activity. Although NF-κB1 and NF-κsites have opposite effects on MLCK promoter activity, it appears that the TNF-α-induced stimulatory effect of NF-κB1 site is greater than the repressive effect of NF-κB2 site, resulting in a net stimulation of MLCK promoter activity.

Although our studies indicated that TNF-α-induced activation of NF-κB leads to an increase in MLCK promoter activity, an interesting question remained as to whether NF-κB activation by other cytokines can also cause an increase in MLCK gene activity. To address this question, we examined the effect of another potent NF-κB activating cytokine IL-1 β on MLCK promoter activity. IL-1 β treatment resulted in a rapid activation of NF-κB and an increase in MLCK promoter activity (unpublished data), suggesting that NF-κB activation by other pro-inflammatory mediators can also induce activation of MLCK gene.

In conclusion, our studies have for the first time identified several molecular determinants that play an essential role in the regulation of basal and TNF-α-stimulated MLCK promoter activity. Our data indicated that MLCK minimal promoter region was located between −313 and +118 on MLCK promoter region. The p53 binding site (−294 to −275) located within the minimal promoter region was an essential determinant for basal promoter activity. The TNF-α-induced increase in MLCK promoter activity was mediated by NF-κB1 site (+48 to +57), and deletion of this site resulted in a complete loss of response to TNF-α treatment. The NF-κB1 and NF-κB2 (−325 to −316) sites have opposite regulatory action on MLCK promoter activity. A likely molecular explanation for the differential effect relates to the differences in the NF-κB dimer type that preferentially binds to the κB sites. The p50/p65 dimer binding to the NF-κB1 site up-regulates the MLCK promoter activity, while p50/p50 binding to the NF-κB2 site down-regulates the promoter activity. These findings provide novel insight into the molecular mechanisms that regulate basal and TNF-α-induced modulation of Caco-2 MLCK gene activity and the subsequent opening of the intestinal epithelial TJ barrier.

## Supplementary Material

The following supplementary material is available for this article:

**Figure SI.** TNF-α effect on cellular expression andactivation of NF-κB subunits in Caco-2 cells. (**A**)TNF-α effect on cellular expression of NF-κB p65 andp50. Caco-2 cells were treated with TNF-α for 30 min. p65 andp50 expressions were determined by Western blot. (**B**)TNF-α effect on NF-κB activation. Caco-2 monolayerswere treated with TNF-α for 30 min; NF-κB p65cytoplasmic-to-nuclear translocation was determined byimmunofluorescent staining with anti-NF-κB p65 antibody.

**Figure S2.** IL-1 β effect on NF-κB activationand MLCK promoter activity. (**A**) IL-1 β (10 ng/ml)effect on NF-κB activation. Caco-2 monolayers were treatedwith IL-1 β for 30 min; NF-κB p65 cytoplasmic-to-nucleartranslocation was determined by immunofluorescent staining withanti-NF-κB p65 antibody. (**B**) IL-1 β (10 ng/ml) effect on MLCK promoter activity.

This material is available as part of the online article from: http://www.blackwell-synergy.com/doi/abs/10.1111/j.1582-4934.2008.00302.x

(This link will take you to the article abstract).

## References

[1] Hollander D, Vadheim CM, Brettholz E, Petersen GM, Delahunty T, Rotter JI (1986). Increased intestinal permeability in patients with Crohn's disease and their relatives. A possible etiologic factor. Ann Intern Med.

[2] Katz KD, Hollander D, Vadheim CM, McElree C, Delahunty T, Dadufalza VD, Krugliak P, Rotter JI (1989). Intestinal permeability in patients with Crohn's disease and their healthy relatives. Gastroenterology.

[3] May GR, Sutherland LR, Meddings JB (1993). Is small intestinal permeability really increased in relatives of patients with Crohn's disease?. Gastroenterology.

[4] Ukabam SO, Clamp JR, Cooper BT (1989). Abnormal small intestinal permeability to sugars in patients with Crohn's disease of the terminal ileum and colon. Digestion.

[5] Bjarnason I, O'Morain C, Levi AJ, Peters TJ (1983). Absorption of 51chromium-labeled ethylened aminetetraacetate in inflammatory bowel disease. Gastroenterology.

[6] Ainsworth M, Eriksen J, Rasmussen JW, Schaffalitzky de Muckadell OB (1989). Intestinal permeability of 51Cr-labelled ethylenedi-aminetetraacetic acid in patients with Crohn's disease and their healthy relatives. Scand J Gastroenterol.

[7] Pironi L, Miglioli M, Ruggeri E, Levorato M, Dallasta MA, Corbelli C, Nibali MG, Barbara L (1990). Relationship between intestinal permeability to [51Cr]EDTA and inflammatory activity in asymptomatic patients with Crohn's disease. Dig Dis Sci.

[8] Munkholm P, Langholz E, Hollander D, Thornberg K, Orholm M, Katz KD, Binder V (1994). Intestinal permeability in patients with Crohn's disease and ulcerative colitis and their first degree relatives. Gut.

[9] Jenkins RT, Ramage JK, Jones DB, Collins SM, Goodacre RL, Hunt RH (1988). Small bowel and colonic permeability to 51Cr-EDTA in patients with active inflammatory bowel disease. Clin Invest Med.

[10] Ma TY (1997). Intestinal epithelial barrier dysfunction in Crohn's disease. Proc Soc Exp Biol Med.

[11] Hollander D (1992). The intestinal permeability barrier. A hypothesis as to its regulation and involvement in Crohn's disease. Scand J Gastroenterol.

[12] Hollander D (1988). Crohn's disease – a permeability disorder of the tight junction?. Gut.

[13] DeMeo MT, Mutlu EA, Keshavarzian A, Tobin MC (2002). Intestinal permeation and gastrointestinal disease. J Clin Gastroenterol.

[14] Bjarnason I, Williams P, Smethurst P, Peters TJ, Levi AJ (1986). Effect of non-steroidal anti-inflammatory drugs and prostaglandins on the permeability of the human small intestine. Gut.

[15] Fasano A, Fiorentini C, Donelli G, Uzzau S, Kaper JB, Margaretten K, Ding X, Guandalini S, Comstock L, Goldblum SE (1995). Zonula occludens toxin modulates tight junctions through protein kinase C-dependent actin reorganization, in vitro. J Clin Invest.

[16] Spitz J, Yuhan R, Koutsouris A, Blatt C, Alverdy J, Hecht G (1995). Enteropathogenic Escherichia coli adherence to intestinal epithelial monolayers diminishes barrier function. Am J Physiol.

[17] Hecht G, Koutsouris A, Pothoulakis C, LaMont JT, Madara JL (1992). Clostridium difficile toxin B disrupts the barrier function of T84 monolayers. Gastroenterology.

[18] Hecht G, Pothoulakis C, LaMont JT, Madara JL (1988). Clostridium difficile toxin A perturbs cytoskeletal structure and tight junction permeability of cultured human intestinal epithelial monolayers. J Clin Invest.

[19] Madara JL (1989). Loosening tight junctions. Lessons from the intestine. J Clin Invest.

[20] Targan SR, Hanauer SB, van Deventer SJ, Mayer L, Present DH, Braakman T, DeWoody KL, Schaible TF, Rutgeerts PJ, Crohn's Disease cA2 Study Group (1997). A short-term study of chimeric monoclonal antibody cA2 to tumor necrosis factor alpha for Crohn's disease. N Engl J Med.

[21] Van Deventer SJ (1997). Tumour necrosis factor and Crohn's disease. Gut.

[22] van Dullemen HM, van Deventer SJ, Hommes DW, Bijl HA, Jansen J, Tytgat GN, Woody J (1995). Treatment of Crohn's disease with anti-tumor necrosis factor chimeric monoclonal antibody (cA2). Gastroenterology.

[23] Hanauer SB, Feagan BG, Lichtenstein GR, Mayer LF, Schreiber S, Colombel JF, Rachmilewitz D, Wolf DC, Olson A, Bao W, Rutgeerts P (2002). Maintenance infliximab for Crohn's disease: the ACCENT I randomised trial. Lancet.

[24] Fiocchi C (2004). Closing fistulas in Crohn's dis-ease– should the accent be on maintenance or safety?. N Engl J Med.

[25] Legnani P, Kornbluth A (2004). Newer Therapies for Inflammatory Bowel Disease. Curr Treat Options Gastroenterol.

[26] Sands BE, Blank MA, Patel K, van Deventer SJ (2004). Long-term treatment of recto-vaginal fistulas in Crohn's disease: response to infliximab in the ACCENT II Study. Clin Gastroenterol Hepatol.

[27] Clayburgh DR, Barrett TA, Tang Y, Meddings JB, Van Eldik LJ, Watterson DM, Clarke LL, Mrsny RJ, Turner JR (2005). Epithelial myosin light chain kinase-dependent barrier dysfunction mediates T cell activation-induced diarrhea in vivo. J Clin Invest.

[28] Clayburgh DR, Musch MW, Leitges M, Fu YX, Turner JR (2006). Coordinated epithelial NHE3 inhibition and barrier dysfunction are required for TNF-mediated diarrhea in vivo. J Clin Invest.

[29] Ferrier L, Mazelin L, Cenac N, Desreumaux P, Janin A, Emilie D, Colombel JF, Garcia-Villar R, Fioramonti J, Bueno L (2003). Stress-induced disruption of colonic epithelial barrier: role of interferon-gamma and myosin light chain kinase in mice. Gastroenterology.

[30] Moriez R, Salvador-Cartier C, Theodorou V, Fioramonti J, Eutamene H, Bueno L (2005). Myosin light chain kinase is involved in lipopolysaccharide-induced disruption of colonic epithelial barrier and bacterial translocation in rats. Am J Pathol.

[31] Suenaert P, Bulteel V, Lemmens L, Noman M, Geypens B, Van Assche G, Geboes K, Ceuppens JL, Rutgeerts P (2002). Anti-tumor necrosis factor treatment restores the gut barrier in Crohn's disease. Am J Gastroenterol.

[32] Ma TY, Iwamoto GK, Hoa NT, Akotia V, Pedram A, Boivin MA, Said HM (2004). TNF-alpha-induced increase in intestinal epithelial tight junction permeability requires NF-kappa B activation. Am J Physiol Gastrointest Liver Physiol.

[33] Wang J, Anders RA, Wang Y, Turner JR, Abraham C, Pfeffer K, Fu YX (2005). The critical role of LIGHT in promoting intestinal inflammation and Crohn's disease. J Immunol.

[34] Schmitz H, Fromm M, Bentzel CJ, Scholz P, Detjen K, Mankertz J, Bode H, Epple HJ, Riecken EO, Schulzke JD (1999). Tumor necrosis factor-alpha (TNFalpha) regulates the epithelial barrier in the human intestinal cell line HT-29/B6. J Cell Sci.

[35] Soderholm JD, Streutker C, Yang PC, Paterson C, Singh PK, McKay DM, Sherman PM, Croitoru K, Perdue MH (2004). Increased epithelial uptake of protein antigens in the ileum of Crohn's disease mediated by tumour necrosis factor alpha. Gut.

[36] Hollander D (2002). Crohn's disease, TNF-alpha, and the leaky gut. The chicken or the egg?. Am J Gastroenterol.

[37] Marano CW, Lewis SA, Garulacan LA, Soler AP, Mullin JM (1998). Tumor necrosis factor-alpha increases sodium and chloride conductance across the tight junction of CACO-2 BBE, a human intestinal epithelial cell line. J Membr Biol.

[38] McKay DM, Singh PK (1997). Superantigen activation of immune cells evokes epithelial (T84) transport and barrier abnormalities via IFN-gamma and TNF alpha: inhibition of increased permeability, but not diminished secretory responses by TGF-beta2. J Immunol.

[39] Rodriguez P, Heyman M, Candalh C, Blaton MA, Bouchaud C (1995). Tumour necrosis factor-alpha induces morphological and functional alterations of intestinal HT29 cl.19A cell monolayers. Cytokine.

[40] Wang F, Graham WV, Wang Y, Witkowski ED, Schwarz BT, Turner JR (2005). Interferon-gamma and tumor necrosis factor-alpha synergize to induce intestinal epithelial barrier dysfunction by up-regulating myosin light chain kinase expression. Am J Pathol.

[41] Ma TY, Boivin MA, Ye D, Pedram A, Said HM (2005). Mechanism of TNF-{alpha} modulation of Caco-2 intestinal epithelial tight junction barrier: role of myosin light-chain kinase protein expression. Am J Physiol Gastrointest Liver Physiol.

[42] Schwarz BT, Wang F, Shen L, Clayburgh DR, Su L, Wang Y, Fu YX, Turner JR (2007). LIGHT signals directly to intestinal epithe-lia to cause barrier dysfunction *via* cytoskeletal and endocytic mechanisms. Gastroenterology.

[43] Fedwick JP, Lapointe TK, Meddings JB, Sherman PM, Buret AG (2005). Helicobacter pylori activates myosin light-chain kinase to disrupt claudin-4 and claudin-5 and increase epithelial permeability. Infect Immun.

[44] Scott KG, Meddings JB, Kirk DR, Lees-Miller SP, Buret AG (2002). Intestinal infection with Giardia spp. reduces epithelial barrier function in a myosin light chain kinase-dependent fashion. Gastroenterology.

[45] Blair SA, Kane SV, Clayburgh DR, Turner JR (2006). Epithelial myosin light chain kinase expression and activity are upregulated in inflammatory bowel disease. Lab Invest.

[46] Ye D, Ma I, Ma TY (2006). Molecular mechanism of tumor necrosis factor-alpha modulation of intestinal epithelial tight junction barrier. Am J Physiol Gastrointest Liver Physiol.

[47] Clayburgh DR, Rosen S, Witkowski ED, Wang F, Blair S, Dudek S, Garcia JG, Alverdy JC, Turner JR (2004). A differentiation-dependent splice variant of myosin light chain kinase, MLCK1, regulates epithelial tight junction permeability. J Biol Chem.

[48] Kutach AK, Kadonaga JT (2000). The downstream promoter element DPE appears to be as widely used as the TATA box in Drosophila core promoters. Mol Cell Biol.

[49] Schmitz ML, Baeuerle PA (1991). The p65 sub-unit is responsible for the strong transcription activating potential of NF-kappa B. EMBO J.

[50] Plaksin D, Baeuerle PA, Eisenbach L (1993). KBF1 (p50 NF-kappa B homodimer) acts as a repressor of H-2Kb gene expression in metastatic tumor cells. J Exp Med.

[51] Udalova IA, Richardson A, Denys A, Smith C, Ackerman H, Foxwell B, Kwiatkowski D (2000). Functional consequences of a polymorphism affecting NF-kappaB p50-p50 binding to the TNF promoter region. Mol Cell Biol.

[52] Grundstrom S, Anderson P, Scheipers P, Sundstedt A (2004). Bcl-3 and NFkappaB p50-p50 homodimers act as transcriptional repressors in tolerant CD4+ T cells. J Biol Chem.

[53] Verma IM, Stevenson JK, Schwarz EM, Van Antwerp D, Miyamoto S (1995). Rel/NF-kappa B/I kappa B family: intimate tales of association and dissociation. Genes Dev.

[54] Hayden MS, Ghosh S (2004). Signaling to NFkappaB. Genes Dev.

[55] Ghosh S, Gifford AM, Riviere LR, Tempst P, Nolan GP, Baltimore D (1990). Cloning of the p50 DNA binding subunit of NF-kappa B: homology to rel and dorsal. Cell.

[56] Kieran M, Blank V, Logeat F, Vandekerckhove J, Lottspeich F, Le Bail O, Urban MB, Kourilsky P, Baeuerle PA, Israel A (1990). The DNA binding subunit of NF-kappa B is identical to factor KBF1 and homologous to the rel oncogene product. Cell.

[57] Nolan GP, Ghosh S, Liou HC, Tempst P, Baltimore D (1991). DNA binding and I kappa B inhibition of the cloned p65 subunit of NF-kappa B, a rel-related polypeptide. Cell.

[58] Shaffer AL, Rosenwald A, Hurt EM, Giltnane JM, Lam LT, Pickeral OK, Staudt LM (2001). Signatures of the immune response. Immunity.

[59] Alizadeh AA, Eisen MB, Davis RE, Ma C, Lossos IS, Rosenwald A, Boldrick JC, Sabet H, Tran T, Yu X, Powell JI, Yang L, Marti GE, Moore T, Hudson J, Lu L, Lewis DB, Tibshirani R, Sherlock G, Chan WC, Greiner TC, Weisenburger DD, Armitage JO, Warnke R, Levy R, Wilson W, Grever MR, Byrd JC, Botstein D, Brown PO, Staudt LM (2000). Distinct types of diffuse large B-cell lymphoma identified by gene expression profiling. Nature.

[60] Keller U, Nilsson JA, Maclean KH, Old JB, Cleveland JL (2005). Nfkb 1 is dispensable for Myc-induced lymphomagenesis. Oncogene.

[61] Ma XY, Wang H, Ding B, Zhong H, Ghosh S, Lengyel P (2003). The interferon-inducible p202a protein modulates NF-kappaB activity by inhibiting the binding to DNA of p50/p65 heterodimers and p65 homod-imers while enhancing the binding of p50 homodimers. J Biol Chem.

[62] Hou S, Guan H, Ricciardi RP (2003). Phosphorylation of serine 337 of NF-kappaB p50 is critical for DNA binding. J Biol Chem.

[63] May MJ, Ghosh S (1997). Rel/NF-kappa B and I kappa B proteins: an overview. Semin Cancer Biol.

[64] Iwamoto GK, Konicek SA (1997). Cytomegalovirus immediate early genes upregulate interleukin-6 gene expression. J Investig Med.

[65] Ma TY, Hollander D, Riga R, Bhalla D (1993). Autoradiographic determination of permeation pathway of permeability probes across intestinal and tracheal epithelia. J Lab Clin Med.

[66] Ma TY, Hollander D, Tran LT, Nguyen D, Hoa N, Bhalla D (1995). Cytoskeletal regulation of Caco-2 intestinal monolayer paracellular permeability. J Cell Physiol.

