# Supplementary figures and images for: Cellular and molecular mechanisms that mediate basal and tumour necrosis factor-α-induced regulation of myosin light chain kinase gene activity
Source: J Cell Mol Med. 2008 Mar 17;12(4):1331–46. doi: 10.1111/j.1582-4934.2008.00302.x (PMC3865676; doi:10.1111/j.1582-4934.2008.00302.x)

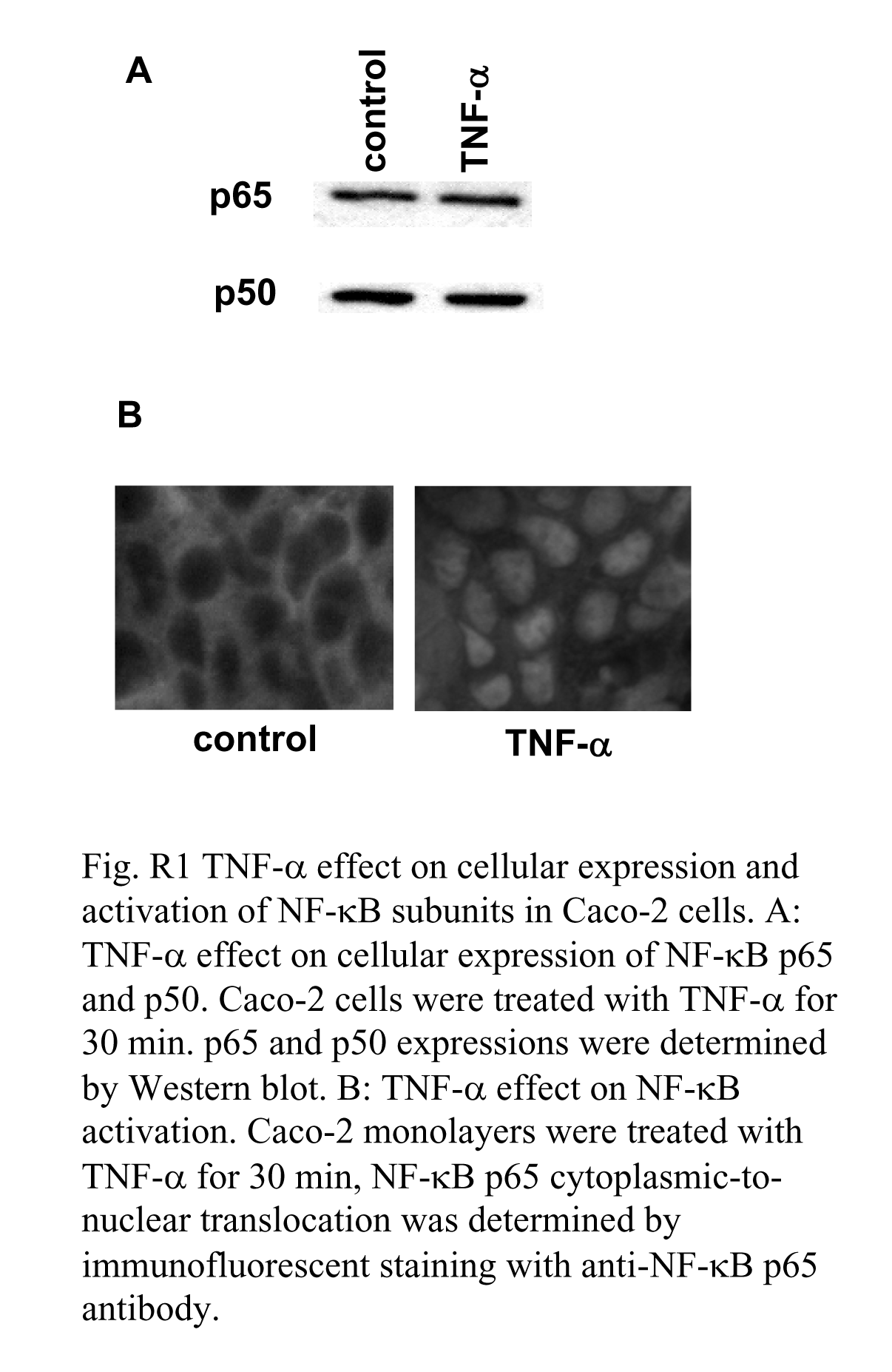

Supplement: Supplementary file 1 [file jcmm0012-1331-SD1.tif]

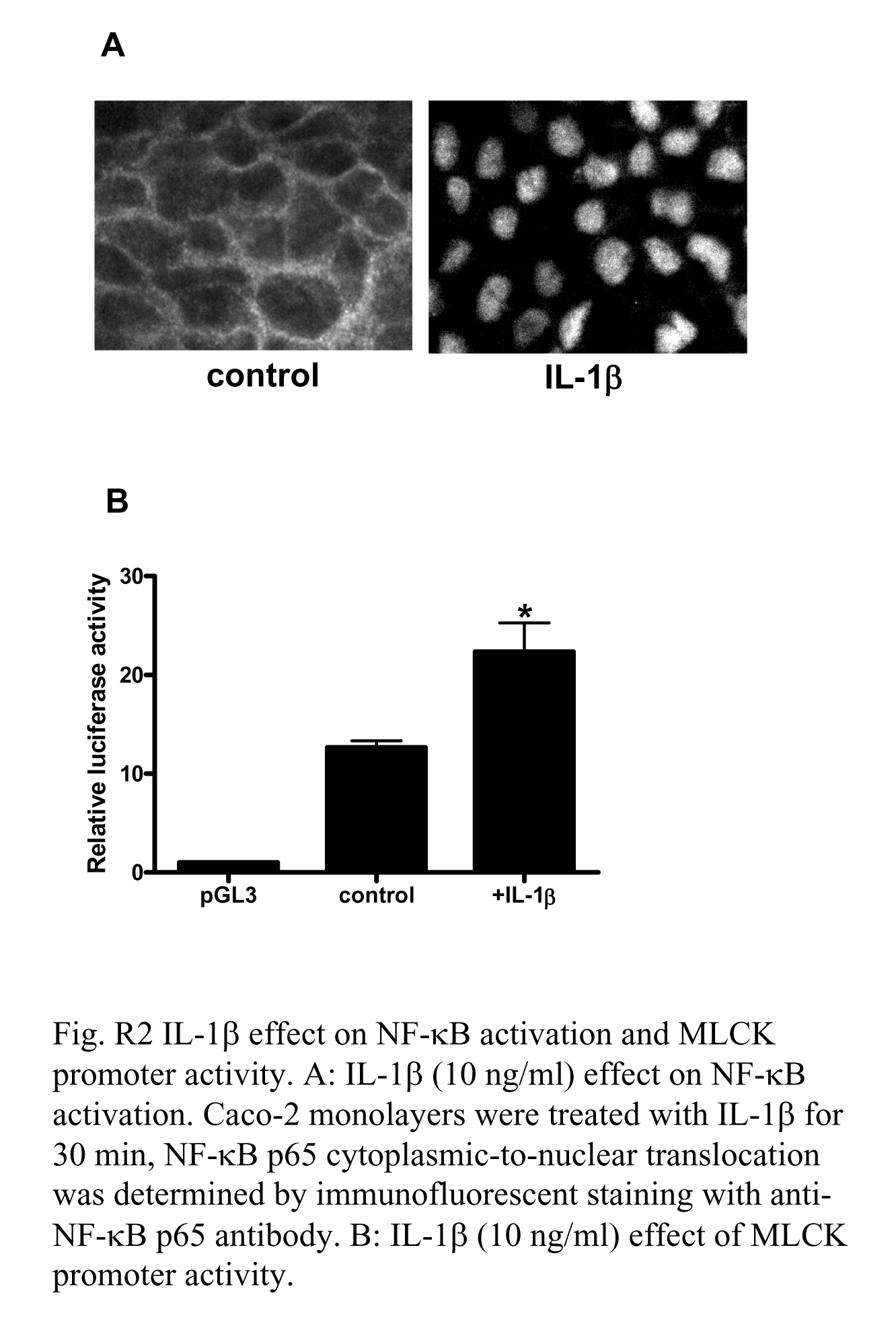

Supplement: Supplementary file 2 [file jcmm0012-1331-SD2.tif]
